# Evaluation of Ab externo subretinal bands removal during pars plana vitrectomy for rhegmatogenous retinal detachment complicated by proliferative vitreoretinopathy

**DOI:** 10.1186/s12886-022-02449-0

**Published:** 2022-05-20

**Authors:** Samir Elbaha, Mohammed Ghoneem, Amir abousamra, Mahmoud Abouhussein

**Affiliations:** Alexandria University, Alexandria, Egypt

**Keywords:** Rhegmatogenous retinal detachment, Pars plana vitrectomy, Subretinal bands

## Abstract

**Background:**

To compare the safety and efficacy of Ab-externo subretinal bands removal in comparison with the classical Ab-interno approach during pars plana vitrectomy for primary rhegmatogenous retinal detachment.

**Methods:**

Subjects aged 28–62 years with primary RRD complicated by proliferative vitreoretinopathy (PVR) with subretinal bands interfering with retinal flattening were treated by pars plana vitrectomy (PPV) and silicone oil injection. Subretinal bands were removed using the classical AB interno approach through one or more retinotomies in ten patients (group A) and using AB externo approach in twenty cases (group B). Post-operative follow-up visits occurred at 1 day, 1 week, 1 month, and 3 months, after surgery. The main outcomes were assessment of subretinal bands removal efficacy, documentation of complications, anatomical reattachment rate, and postoperative best-corrected visual acuity (BCVA).

**Results:**

There was no statistically significant difference between both groups regarding patients’ age, gender, lens status, and the onset of retinal detachment. Seventy percent of both groups presented with inferior retinal detachment while ten percent presented with temporal detachments and twenty percent had a total retinal detachment. Both groups had a statistically significant improvement in postoperative visual acuity in comparison with preoperative visual acuity (*P* = 0.005 for group A and *P* =  < 0.001 for group B). There was no statistically significant difference between both groups regarding preoperative (*P* = 0.928) and postoperative (*P* = 0.185) visual acuity. A higher incidence of complications was reported in group A (40%) in comparison with group B (30%) but this difference was not statistically significant (*P* = 0.69). More Epimacular membranes were seen postoperatively in group A (30%) in comparison with group B (20%) but again this difference was not statistically significant (*P* = 0.657). Subretinal hemorrhage was seen in ten percent of cases in both groups. Intraocular pressure was measured in every follow-up of all patients in both groups, no statistically significant difference was found between both groups.

**Conclusions:**

Both techniques are effective and safe to remove subretinal bands with similar outcomes.

**Supplementary Information:**

The online version contains supplementary material available at 10.1186/s12886-022-02449-0.

## Background

PVR is a scarring process developing in a portion of retinal detachment cases and is the most common cause of surgical failure of RRD. The growth and contraction of membranes are the hallmarks of PVR. Contraction may develop on either side of the retina or in the retinal tissue itself [1]. It has been reported that 5% to 10% of all retinal detachments develop PVR [2].

The exact pathogenesis of PVR is not fully understood to date. It is believed that a process called epithelial-mesenchymal transition (EMT) of the retinal pigment epithelium might be involved in the PVR formation process [3].

Takayama et al. demonstrated the involvement of miR-148a-3p in the regulation of the migration ability of RPE cells. Toro et al. identified more than 20 miRNAs in vitreous humor samples of patients with different grades of PVR. Increased expression of miRNA was seen in samples of worse PVR cases [3].

Pietras-Baczewska et al. studied the antioxidants levels in the vitreous humor of eyes with and without PVR. No statistical differences between groups in total antioxidants level (TAS values) (*p* = 0.81) were found. However, TAS levels were higher in samples of patients with a longer duration of retinal detachment with PVR. High oxidative stresses were proven to cause more damage to the retina by accelerating apoptosis to photoreceptors and ganglion cells [4].

A widely accepted classification of PVR is the one developed by the Retina Society Terminology Committee in 1983 [5]. This classification has several limitations including the absence of consideration for the location of the vitreoretinal traction ring and magnitude of contraction to the vitreous base [1]. A revised classification was developed in 1991 taking into account the location, severity, and extent of PVR [6].

Grade A is the earliest grade of PVR. Pigment clumps can be seen on the inferior retina or in the vitreous cavity. These are believed to be a result of pigment cell multiplication. A decreased vitreous mobility is seen in this early grade [6].

Grade B is characterized by rigid appearing retinal surface, tortuous retinal vessels, and inner retinal wrinkling (including rolled edges of retinal breaks). These changes are due to thin epiretinal membranes [6].

Full-thickness retinal folds are seen in grade C. The updated classification further subdivided this grade into posterior (P) and anterior (A) forms according to the location of folds roughly anterior and posterior to the equator of the globe. The severity of this grade is expressed according to the number of clock hours of the retina involved (1–12) [6].

The location of the cellular proliferation will determine the clinical presentation of PVR. For example, if the cellular activity is present on the inner retina, membrane contraction can manifest as a retinal folding or distortion known as a star fold. If cellular proliferation is present in the subretinal space, fibrous strands or irregular plaques are seen [2].

The risk factors for developing different types of PVR are not well defined. Regression analysis showed a positive correlation between developing subretinal PVR with more retinal quadrants detached, longer duration of RD, atrophic retinal breaks, and younger age [7].

Pars plana vitrectomy is a technique of choice of many surgeons nowadays for the treatment of RRD. Vitrectomy is necessary for many situations such as tractional retinal detachments, RRD complicated by PVR, and if the surgical view is hindered by vitreous hemorrhage or dense cataract. The procedure involves the removal of vitreous humor and substituting it with gas or liquid tamponade to seal breaks. During the procedure, breaks are treated by either laser photocoagulation or cryopexy [8].

Steve Charles developed the punch-through technique for the removal of subretinal bands. A pair of 25-gauge retinal forceps is pushed through the retina with the blades closed to access the subretinal space at a point just adjacent to the subretinal band. The blades are then opened to grasp and pull the subretinal band out of the retinotomy created. The endoiluminator can also be used in this technique to act as a fulcrum to increase the tangential pull on the band. The subretinal bands could also be accessed through the original retinal breaks if possible and removed using retinal forceps. Often subretinal bands are dense and fibrous and couldn’t be removed safely, in such situations dividing them with scissors through a retinotomy would be enough to relieve the traction [9].

The intraoperative use of intravitreal triamcinolone or Dexamethasone implant was studied by Mirshahi et al. and Banerjee et al. respectively aiming to prevent postoperative PVR, but the results are still controversial, and no consensus exists on their use [10].

In 2015 Bernard Wolff developed a new technique for subretinal bands removal through an Ab Externo approach during a scleral buckling operation. The subretinal bands are removed through a 23- or 25-gauge valved cannula placed in the subretinal space through the sclera. First, the posterior segment is visualized by an endoilluminator with the help of a non-contact viewing system mounted on an operating microscope. The area of highest detachment is identified and the trocar is advanced at a 15-degree angle to pierce the sclera and choroid in this area. The cannula is then visualized to be placed in the subretinal space and retinal forceps are advanced through it to access the subretinal band. The band is then grasped and pulled out of the eye. A flute needle is then placed in the subretinal space to drain subretinal fluid controllably and the rest of the surgery is completed in the standard fashion [11].

This novel technique avoids the need for creating a separate retinotomy for subretinal bands removal. A retinotomy may increase the risk of developing epiretinal membranes as shown by a recent study published in the American Journal Of Ophthalmology [12]. While potential complications with the Ab Externo approach include retinal injury and subretinal bleeding [11].

Our work aimed to evaluate the efficacy and safety of ab-externo subretinal bands removal in comparison with the classical ab-interno approach during vitrectomy for primary rhegmatogenous retinal detachment.

## Methods

### Patients and study design

The study was conducted on thirty cases of primary RRD in the Alexandria Main University Hospital, Alexandria, Egypt. All patients had RRD complicated by subretinal bands interfering with retinal flattening. Patients with recurrent RD, non-rhegmatogenous RD, combined rhegmatogenous and choroidal detachments, and shallow RD were excluded. Eyes, where subretinal bands cannot be visualized preoperatively, were not included in our study. Cases were distributed randomly into two groups with a randomization ratio (2:1); ten cases underwent pars plana vitrectomy with subretinal bands removal using the classical Ab-interno approach through one or more retinotomies (group A) and twenty cases underwent pars plana vitrectomy with subretinal bands removal through the ab-externo approach (group B).

A detailed history was taken from all patients including patients’ age and gender, duration of retinal detachment, and any relevant medical history. The preoperative ocular examination included uncorrected and best-corrected visual acuity, anterior segment examination, and intraocular pressure (IOP) measurement by applanation tonometry. Fundus examination was done with detailed documentation of retinal detachment configuration and location of breaks.

Alexandria University ethics review board approval was obtained and the research followed the tenets of the Declaration of Helsinki. Informed consent was taken from all patients participating in the study after explaining patients’ current condition, the surgery needed, benefits, and possible complications of the surgical intervention.

All procedures were done under general anesthesia and informed consent was taken from all patients and any risks involved were explained thoroughly.

## Surgical technique

### Group A

Three valved 23G cannulas are inserted through pars plana at 3.5 mm from the limbus in pseudophakic patients and 4 mm from the limbus in phakic patients. One cannula at the inferotemporal position, one at the superotemporal position, and one at the superonasal position. The posterior segment is then visualized using the endoluminator as a light source and a wide-field Resight (Carl Zeiss Meditec AG) noncontact viewing system mounted on an operating microscope. Vitrectomy is completed in a standard fashion using the Constellation vitrectomy system (Alcon Laboratories, Inc, Fort Worth, TX). Core vitrectomy, posterior vitreous detachment, vitreous base shaving, and identification of breaks. A small retinotomy is created at the highest point of retinal elevation caused by the subretinal band at a point just adjacent to the band [5]. A 23G forceps are introduced through the retinotomy and then opened to grasp, lift, and remove the band [5]. Fluid air exchange, flattening of the retina, and silicone oil (2000 cst) injection are then carried out. Pars plana cannulas are then removed and sclerotomies sutured if needed.

### Group B

**Fig. 1:**
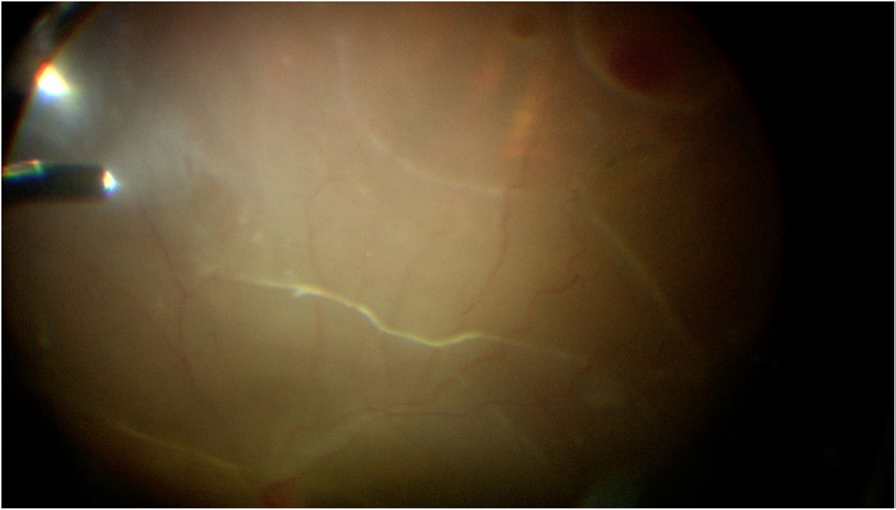
Surgical view of the posterior segment in a case of RRD with subretinal bands using a non-contact viewing system on a surgical microscope

**Fig. 2:**
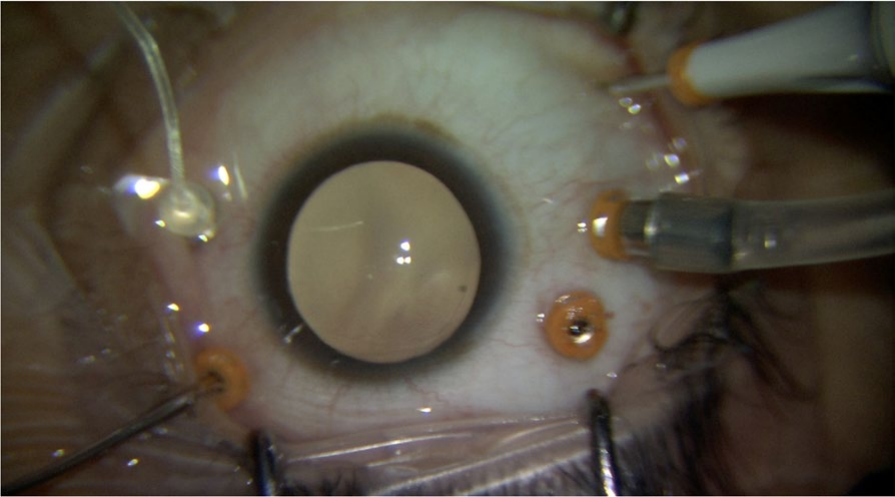
Surgical view of the anterior segment while insertion of the 23G trocar into the subretinal space

**Fig. 3:**
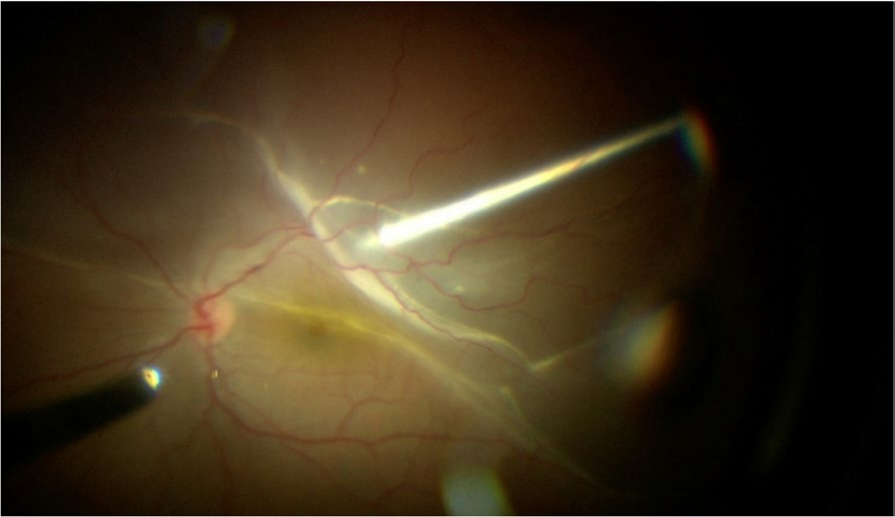
A pair of retinal forceps is advanced through the subretinal cannula and is used to grasp the subretinal band and extract it out of the eye

Three valved 23G cannulas are inserted through pars plana at 3.5 mm from the limbus in pseudophakic patients and 4 mm from the limbus in phakic patients. One cannula at the inferotemporal position, one at the superotemporal position, and one at the superonasal position. Infusion line secured at inferotemporal cannula but turned off. A 25G chandelier is inserted at the inferonasal position. Using the endoluminator the cannula is visualized to be passing through the pars plana and not in subretinal space. The posterior segment is then visualized using the endoluminator as a light source and a wide-field Resight (Carl Zeiss Meditec AG) non-contact viewing system mounted on an operating microscope. The most elevated point of the retinal detachment is identified and an appropriate external position for access to the subretinal band is chosen (Fig. 1). During internal visualization, a 23G trocar is advanced at an angle of around 15°, penetrating the sclera and the choroid, and entering the subretinal space (Fig. 2). Once it has been visually confirmed that the cannula entered the subretinal space, the trocar is removed and the cannula remains in the subretinal space [6]. Vitreoretinal forceps are then introduced through the valved cannula to grab and remove subretinal membranes through the cannula [6]. (Fig. 3). After complete membrane removal, the cannula can be removed and balanced salt solution infusion is switched on [6]. Vitrectomy is then completed in a standard fashion using the Constellation vitrectomy system (Alcon Laboratories, Inc, Fort Worth, TX). Core vitrectomy, posterior vitreous detachment, vitreous base shaving, identification of breaks, fluid air exchange, flattening of the retina, and silicone oil injection (2000 cst). Pars plana cannulas are then removed and sclerotomies sutured if needed. None of our patients required a combined phacoemulsification and vitrectomy procedure.

Patients in both groups received appropriate postoperative treatment. Patient follow-up was carried out at 1 day, 1 week, 1 month, and 3 months intervals. The following criteria were assessed: Efficacy in the removal of subretinal bands, complication documentation such as subretinal or vitreous hemorrhage, retinal perforation, and intraoperative hypotony, anatomical success in treating retinal detachment, and functional success regarding final postoperative visual acuity gain.

### Sample size and statistical analysis

 Data were fed to the computer and analyzed using IBM SPSS software package version 20.0. (Armonk, NY: IBM Corp). Qualitative data were described using numbers and percentages. Shapiro–Wilk test was used to verify the normality of distribution. Quantitative data were described using range (minimum and maximum), mean, standard deviation, median, and interquartile range (IQR). The significance of the obtained results was judged at the 5% level. The used tests were, the Chi-square test for categorical variables, to compare between different groups, Fisher’s Exact or Monte Carlo correction for chi-square when more than 20% of the cells have an expected count of less than 5, Mann Whitney test for abnormally distributed quantitative variables, to compare between two studied groups and Wilcoxon signed ranks test for abnormally distributed quantitative variables, to compare between two periods.

## Results

Table 1 shows the comparison between groups A and B according to age. Regarding patients’ ages, group A ranged from thirty-two to sixty-two while group B ranged from twenty-eight to sixty-one. The mean age was 45.33 ± 8.93 in group A while was 46.53 ± 10.29 in group B. there was no significant difference between both groups regarding patients’ age (*P* = 0.736).

**Table 1:** Comparison between both groups regarding patients’ age

	**Group A (** ***n*** ** = 10)**	**Group B (** ***n*** ** = 20)**	**Test of sig**	***p***
**Age (years)**				
Min. – Max	32.0 – 62.0	28.0 – 61.0	t = 0.341	0.736
Mean ± SD	45.33 ± 8.93	46.53 ± 10.29		
Median	43.0	47		

Group A had seven males (70%) and three females (30%) while group B had eleven males (55%) and nine females (45%). There was no significant difference between the two groups regarding gender (*P* = 0.694).

Table 2 shows the difference between both groups regarding lens status. Seven patients (70%) were phakic in group A while only three (30%) were pseudophakic. On the other hand, twelve patients (60%) were phakic in group B and eight (40%) were pseudophakic. There was no statistically significant difference between both groups regarding lens status (*P* = 0.702).

**Table 2:** Comparison between both groups regarding gender, lens status, duration of retinal detachment, and retinal detachment configuration

	**Group A (** ***n*** ** = 10)**		**Group B (** ***n*** ** = 20)**		**Test of sig**	***p***
	**No**	**%**	**No**	**%**		
**Gender**						
Male	7	70.0	11	55.0	χ^2^=0.625	0.694
Female	3	30.0	9	45.0		
**Lens status**						
Phakic	7	70	12	60	0.287	0.702
Pseudophakic	3	30	8	40		
**Duration of detachment (months)**						
Min. – Max	2.0 – 24.0	2.0 – 24.0	2.0 – 24.0	2.0 – 24.0	U = 77.50	0.314
Mean ± SD	11.70 ± 7.24	9.10 ± 6.23	9.10 ± 6.23	9.10 ± 6.23		
Median (IQR)	11.0 (6.0 – 12.0)	9.0 (4.5 – 12.0)	9.0 (4.5 – 12.0)	9.0 (4.5 – 12.0)		
**Retinal detachment configuration**						
Inferior	7	70.0	14	70.0	0.294	1.00
Temporal	1	10.0	2	10.0		
Total	2	20.0	4	20.0		

Table 2 demonstrates the duration of retinal detachment in months in both groups. Both groups had retinal detachment symptoms ranging from two to twenty-four months. Group A had a higher mean of 11.70 ± 7.24 months compared to group B’s mean of 9.10 ± 6.23 months. There was no statistically significant difference between both groups (*P* = 0.314).

Inferior retinal detachment was the most common configuration in both groups (70%) followed by total retinal detachment (20%) and then followed by temporal configuration (10%).

Table 3 show the comparison between both groups regarding preoperative and final postoperative visual acuities (at 3 months) in decimal. Group B had a slightly better preoperative visual acuity (mean 0.027 ± 0.030 in decimal and –1.98 ± 0.75 in logMAR) in comparison with group A (mean 0.026 ± 0.031 in decimal and –1.99 ± 0.76). On the other hand group A had a better postoperative visual acuity (mean 0.130 ± 0.063 in decimal and –0.94 ± 0.24 in logMAR) in comparison with group B (mean 0.101 ± 0.055 in decimal and 1.06 ± 0.25 in logMAR). There was no statistically significant difference between both groups regarding both preoperative and postoperative visual acuities (*P* = 0.928 and *P* = 0.185 respectively).

**Table 3:** Comparison between both groups regarding preoperative and postoperative visual acuity

VA in decimal	Group A (*n* = 10)	Group B (*n* = 20)	U	*p*
**Preoperative**				
Min. – Max	0.001 – 0.100	0.001 – 0.100	98.0	0.928
Mean ± SD	0.026 ± 0.031	0.027 ± 0.030		
Median (IQR)	0.020 (0.001 – 0.030)	0.020 (0.001 – 0.040)		
**Postoperative**				
Min. – Max	0.050 – 0.200	0.030 – 0.200	71.0	0.185
Mean ± SD	0.130 ± 0.063	0.101 ± 0.055		
Median (IQR)	0.100 (0.100 – 0.200)	0.100 (0.050 – 0.160)		
**p**_**1**_	**0.005** ^*^	** < 0.001** ^*^		

There was a statistically significant improvement in visual acuity in the postoperative period in comparison with the preoperative period in both groups. *P*-value was 0.005 for group A and was < 0.001 for group B.

Table 4 shows the comparison between both groups according to the frequency of complications encountered. A higher frequency of complications was seen in group A (40%) in comparison with group B (30%). This difference was not statistically significant (*P* = 0.690).

**Table 4:** Comparison between both groups regarding complications

**Complications**	**Group A** **(** ***n*** ** = 10)**		**Group B** **(** ***n*** ** = 20)**		${{\varvec{\chi}}}^{2}$	^**FE**^ **p**
	**No**	**%**	**No**	**%**		
Negative	6	60.0	14	70.0	0.300	0.690
**Positive**	**4**	**40.0**	**6**	**30.0**		
Epimacular membrane	3	30.0	4	20.0	0.373	0.657
Subretinal hemorrhage	1	10.0	2	10.0	0.000	1.000

Epimacular membrane formation was a more commonly encountered complication in both groups followed by subretinal hemorrhage. Thirty percent of patients in group A developed epimacular membranes while only ten percent had a subretinal hemorrhage. Twenty percent of patients in group B developed epimacular membranes but only ten percent had a subretinal hemorrhage.

More epiretinal membranes were seen in group A (30%) in comparison to group B (20%). This difference was not statistically significant (*P* = 0.657). Ten percent of patients in both groups had subretinal hemorrhage (*P* = 1.0).

Two cases were complicated by subretinal hemorrhage in group B and were encountered intraoperatively during trocar insertion. Fortunately, the hemorrhage was minimal and did not obscure visualization of the subretinal band. The hemorrhage was left to be resolved on its own during the postoperative period.

One case was complicated by a subretinal hemorrhage in group A and was again encountered intraoperatively. The hemorrhage occurred when the end gripping forceps inadvertently touched the choroid while being introduced through the retinotomy to grasp the subretinal band. Immediately the infusion pressure was raised to try and control the bleeding. Fortunately, the hemorrhage was controlled and a minimal amount of blood was aspirated through the retinotomy.

All cases in both groups were silicone-filled at the end of the three months follow-up period. The seven cases in both groups who developed epimacular membranes will be managed by epimacular membrane removal at the time of silicone oil removal.

Table 5 shows the difference between both groups regarding postoperative intraocular pressure at different follow-up periods. Group A had a higher mean postoperative Intraocular pressure at one day, one week, one month, and three months (17.20 ± 4.83, 14.0 ± 2.83, 14.60 ± 3.66, 14.40 ± 2.27 respectively) in comparison with group B mean postoperative intraocular pressure (17.10 ± 4.54, 13.75 ± 2.53, 14.45 ± 3.75, 14.20 ± 2.14 respectively), but the difference was not statistically significant (*P* = 0.956, 0.808, 0.918, 0.815 respectively). Patients who had intraocular pressure above 20 in any of the postoperative periods were treated with topical intraocular pressure-lowering medications. All patients with high intraocular pressure responded well to medical treatment and none required extra interventions.

**Table 5:** Intraocular pressure readings in both groups along the follow-up period

Intraocular pressure	Group A (*n* = 10)	Group B (*n* = 20)	t	*p*
**1 day**				
Min. – Max	14.0 – 26.0	13.0 – 27.0	0.056	0.956
Mean ± SD	17.20 ± 4.83	17.10 ± 4.54		
Median (IQR)	15.0 (14.0 – 18.0)	15.0 (14.0—18.0)		
**1 week**				
Min. – Max	10.0 – 18.0	10.0 – 18.0	0.245	0.808
Mean ± SD	14.0 ± 2.83	13.75 ± 2.53		
Median (IQR)	13.0 (12.0 – 16.0)	13.0 (12.0 – 16.0)		
**1 month**				
Min. – Max	12.0 – 22.0	11.0 – 23.0	0.104	0.918
Mean ± SD	14.60 ± 3.66	14.45 ± 3.75		
Median (IQR)	13.0 (12.0 – 16.0)	13.50 (12.0 – 15.0)		
**3 months**				
Min. – Max	12.0 – 18.0	11.0 – 18.0	0.236	0.815
Mean ± SD	14.40 ± 2.27	14.20 ± 2.14		
Median (IQR)	14.0 (12.0 – 16.0)	14.0 (12.0 – 15.50)		

## Discussion

Our study was a randomized prospective study performed on thirty primary rhegmatogenous retinal detachment patients presenting with subretinal bands. Ten patients were assigned to group A where subretinal bands were removed by the classical Ab-interno approach and the remaining twenty patients were assigned to group B and subretinal bands removed using the ab-externo approach. We excluded patients with non-rhegmatogenous retinal detachments, choroidal detachments, and patients with shallow retinal detachment.

There was no statistically significant difference between both groups regarding patients’ age, gender, lens status, and the onset of retinal detachment. Seventy percent of both groups presented with inferior retinal detachment while ten percent presented with temporal detachments and twenty percent had a total retinal detachment.

Both groups had a statistically significant improvement in postoperative visual acuity in comparison with preoperative visual acuity (*P* = 0.005 for group A and *P* =  < 0.001 for group B). There was no statistically significant difference between both groups regarding preoperative (*P* = 0.928) and postoperative (*P* = 0.185) visual acuity.

A higher incidence of complications was reported in group A (40%) in comparison with group B (30%) but this difference was not statistically significant (*P* = 0.69). More epimacular membranes were seen postoperatively in group A (30%) in comparison with group B (20%) but again this difference was not statistically significant (*P* = 0.657). Subretinal haemorrhage was seen in ten percent of cases in both groups. Intraocular pressure was measured in every follow-up of all patients in both groups, no statistically significant difference was found between both groups.

W.fan and colleagues studied trans-scleral incision for peeling of subretinal proliferation in the setting of pars plana vitrectomy. The technique they used to remove the subretinal bands was similar to the technique we used in our study with the difference that the subretinal bands were removed after the vitrectomy was completed [13]. In our study we removed the subretinal bands before vitrectomy was initiated, we believe that this would be safer to avoid retinal incarceration that might occur from minimal fluid leakage from the subretinal cannula creating a pressure gradient pushing the retina towards the subretinal cannula. eye. We also believe that insertion of an extra trocar and cannula in the subretinal space after completion of vitrectomy would be technically difficult compared to a well-pressurized eye at the beginning of the surgery. In our experience, during vitrectomy, the thick subretinal fluid is frequently evacuated and the retina, as a result, becomes closer to the choroid making it more difficult and riskier to remove subretinal bands through an ab-externo approach. For these reasons, we recommend an ab-externo subretinal bands removal before initiating vitrectomy contrary to the W.fan technique.

W.fan study was conducted on 8 cases with grade C PVR. The mean preoperative and postoperative visual acuities were 2.52 ± 0.18 and 1.80 ± 0.34 in log MAR respectively [13]. Our study included more cases (20 eyes) undergoing Ab-externo subretinal bands removal. We report netter mean preoperative and postoperative visual acuities were 1.98 ± 0.75 and 1.06 ± 0.25 respectively. This difference could be attributed to the better mean preoperative visual acuity in our study.

No subretinal hemorrhage nor Epiretinal membranes were reported in the W.fan study [13]. In our study we encountered 2 eyes complicated by minimal subretinal hemorrhage and 4 cases with epimacular membranes. A higher complication rate could be explained due to the higher sample size in our study.

## Conclusion

Despite the Ab-externo approach being more technically challenging, we concluded that both techniques are safe and effective in subretinal bands removal in the setting of pars plana vitrectomy. Both techniques had similar outcomes with no statistically significant difference regarding complications. We believe the Ab-externo approach is best for cases with high retinal detachment thus making manipulation in the subretinal space easier and safer. If retinal detachment is shallow, we believe the classical method is a safer option.

## Supplementary Information

**Additional file 1.**

## Data Availability

Datasets generated and analyzed during the current study are not publicly available due to confidentiality issues but are available from the corresponding author on reasonable request.
